# A Sema3C Mutant Resistant to Cleavage by Furin (FR-Sema3C) Inhibits Choroidal Neovascularization

**DOI:** 10.1371/journal.pone.0168122

**Published:** 2016-12-30

**Authors:** Shira Toledano, Huayi Lu, Agustina Palacio, Keren Ziv, Ofra Kessler, Shlomit Schaal, Gera Neufeld, Yoreh Barak

**Affiliations:** 1 Cancer Research and Vascular Biology Center, The Bruce Rappaport Faculty of Medicine, Technion, Israel Institute of Technology, Haifa, Israel; 2 Second Hospital of Jilin University, Changchun, Jilin Province, P.R. China; 3 Department of Ophthalmology and Visual Sciences, University of Louisville, Louisville, Kentucky, United States of America; 4 Department of Ophthalmology and Visual Sciences, University of Massachusetts School of Medicine, Massachusetts, United States of America; 5 Department of Ophthalmology, RAMBAM Medical Center, Haifa, Israel; University of Miami, UNITED STATES

## Abstract

In age-related macular degeneration (AMD), abnormal sub retinal choroidal neovascularization (CNV) is a major cause of blindness. FR-sema3C is a point mutated form of semaphorin-3C that is resistant to cleavage by furin like pro-protein convertases (FPPC). We have found in previous work that FR-sema3C functions as an anti-angiogenic factor. In this study we investigated the possible use of FR-sema3C as an inhibitor of CNV. FR-sema3C inhibits VEGF as well as PDGF-BB signal transduction in endothelial cells and to less extent bFGF induced signal transduction using a mechanism that does not depend upon the binding of VEGF like the drugs that are currently the mainstay treatment for AMD. CNV was induced in eyes of C57 black mice by laser photocoagulation. Intravitreal injection of FR-Sema3C or aflibercept (VEGF-trap) was then used to inhibit CNV formation. Invading choroidal vessels were visualized a week later by injection of FITC-dextran into the circulation, followed by the measurement of the area of the invading blood vessels. Injection of 0.1 μg FR-Sema3C inhibited CNV by 55% (P<0.01) and was as effective as 5 μg aflibercept. FR-sema3C did not display any adverse effects on retinal function following its injection into eyes of healthy mice as assessed by optokinetic reflex (OKR) and Electro-retinogram (ERG) criteria. Furthermore, FR-sema3C did not induce apoptosis in the retina as determined by TUNEL nor was there any discernable structural damage to the retina as assessed by several immuno-histochemical criteria. Our results suggest that FR-sema3C could perhaps be used for the treatment of AMD, and that it may perhaps be of benefit to patients that do not respond well to current treatments relying on VEGF sequestering agents.

## Introduction

Age-related macular degeneration (AMD) is the leading cause of blindness in elderly patients in developed countries. Although both forms of AMD (geographic atrophy or dry AMD and choroidal neovascularization or exudative AMD) affect central vision, the exudative form poses the greatest risk for severe visual loss because it progresses much faster. The exudative form is relatively fast progressing and is characterized by choroidal neovascularization (CNV), a process in which new leaky blood vessels originating in the choroid invade the retina [1,2].

Among the angiogenic factors investigated in CNV formation, VEGF was found to be a key factor in animal models [3] and human exudative AMD patients [4], although additional angiogenic factors such as basic fibroblast growth factor (bFGF) and platelet derived growth factor (PDGF) may play a role as well [5,6]. VEGF inhibitors such as bevacizumab (Avastin^™^), ranibizumab (Lucentis^™^) or aflibercept (Eylea^™^) have proven to be effective in treating exudative AMD and represent the current mainstay therapeutic treatment [7]. However, the magnitude of non-response to treatment, defined as no improvement in visual acuity or in reading ability, is substantial, as evidenced by recently published large clinical prospective randomized trials. Thus in the Comparison of Age-Related Macular Degeneration Treatments Trial (CATT), more than 12% of patients lost more than 5 letters and more than 30% of patients experienced no significant change or suffered deterioration in visual acuity despite intensive and regular intra-vitreal injections of either bevacizumab or ranibizumab [8]. Similarly, in the Anti-VEGF Antibody for the treatment of predominantly classic choroidal neovascularization (ANCHOR) clinical trial, 22% of patients treated with monthly ranibizumab injections experienced no significant change or suffered deterioration in visual acuity [9]. Smaller prospective studies concluded that 45% of patients treated with intravitreal bevacizumab were non-responders [9]. Furthermore, patients treated repetitively with anti-VEGF medication may be prone to develop geographic macular atrophy [8]. There are also indications suggesting that patients develop resistance to treatment over time [10]. Thus, novel pharmacotherapy based on non-VEGF targeted mechanisms is required for better control of this disease.

The class-3 semaphorins include seven secreted semaphorins of which six (with the exception of sema3E) utilize either neuropilin-1 or neuropilin-2 or both as their binding receptors [11,12]. The intracellular domains of the neuropilins are short and therefore the neuropilins cannot transduce semaphorin signals on their own and must associate with various receptors of the plexin family [11,13,14]. Interest in a potential role for these semaphorins in the regulation of angiogenesis was kindled when it was found that neuropilins also function as receptors for VEGF family members [15,16]. These observations lead to the characterization of several class-3 semaphorins such as sema3F, sema3A and sema3E as potent inhibitors of angiogenesis [17–19] and to the characterization of several semaphorins as inhibitors of pathological retinal angiogenesis [20–23] and laser photocoagulation induced CNV [23,24]. In view of these observations anti-angiogenic class-3 semaphorins would have been expected to function exclusively as inhibitors of tumor progression. However, several class-3 semaphorins have been found to display mixed functions. Thus, sema3A can also induce recruitment of endothelial progenitor cells and thus also promote angiogenesis [25] and sema3E is cleaved by furin like pro-protein convertases (FPPC) which are generally strongly up-regulated in tumors [26] to generate a major peptide that displays potent pro-tumorigenic properties [19].

Unlike most of the other class-3 semaphorins, Sema3C was initially characterized as a pro-tumorigenic semaphorin [27,28] and it was even thought that it may function as an angiogenic factor [29]. However, it was recently found that contrary to previous reports, sema3C functions as a potent anti-angiogenic agent that potently inhibits retinal oxygen induced angiogenesis [23] as well as tumor angiogenesis [30]. sema3C, like the other class-3 semaphorins, contains two conserved cleavage sites for FPPC [31]. Following cleavage by FPPC, sema3C losses its ability to induce the collapse of the cytoskeleton of responsive cells such as vascular endothelial cells [30]. Surprisingly, we have recently obtained evidence suggesting that one of the cleavage products, p65-Sema3C, which we previously thought was completely inactive, actually functions as a survival/proliferation factor for lung cancer cells [30]. It is thus possible that p65-Sema3C generated by FPPC cleavage rather than full length sema3C is responsible for the reported pro-tumorigenic properties of sema3C while full length sema3C may function as an inhibitor of tumor angiogenesis and tumor progression like the rest of the class-3 semaphorins [32]. Indeed, we have found that a point mutated full length furin resistant sema3C (FR-sema3C) also displays potent anti-angiogenic as well as anti-lymphangiogenic activities [30]. The anti-angiogenic effects of sema3C seem to be mediated by the neuropilin-1 and plexin-D1 receptors [23] while the effects on lymphangiogenesis were mediated by the neuropilin-2, plexin-A1 and plexin-D1 receptors [30] which probably form a complex that constitutes the functional receptor of sema3C as shown for other class-3 semaphorin receptors [33].

These studies suggest that FR-sema3C could be used as an anti-angiogenic drug for the treatment of diseases associated with pathological angiogenesis, and that it may be preferable to wild type sema3C which is susceptible to cleavage by FPPC and may thus produce unwanted side effects. In the present study we show that CNV induced by laser photocoagulation in a mouse model can be inhibited efficiently by intra-vitreal injection of FR-sema3C at a concentration 50 fold lower than the concentration that produced a similar inhibitory effect of aflibercept. Finally, this study demonstrates that injection of FR-sema3C into untreated mouse eyes is not accompanied by loss of retinal function as determined by OKR and ERG tests.

## Methods

### Materials

Recombinant VEGF_165_ and bFGF were produced and purified as previously described [34,35]. Recombinant PDGF-BB, recombinant HGF and recombinant EGF were purchased (Recombinant human PDGF-BB, recombinant human HGF and recombinant human EGF, PeproTech, Rocky Hill, New Jersey, USA). Human FR-sema3C was produced as previously described [30]. The elution buffer (100 mM glycine, 24 mM Tris, pH-7.2) was injected as vehicle in in-vivo experiments. Recombinant human Sema-3C was purchased from R&D systems, Minneapolis, Minnesota, USA. Aflibercept (Eylea^™^) was purchased from Regeneron pharmaceuticals, Inc., Tarrytown, New York, USA. Anti-phospho-ERK1/2 (sc-7383), anti-sema3C (sc-27796), anti-total ERK1/2 (sc-153), anti-Akt (sc-5298), anti-pFAK^125^ (Tyr397) (sc-11765) and anti-FAK^125^ antibodies (sc-932) were from Santa Cruz Biotechnology, Dallas, Texas U.S.A. Anti-Phospho-p38 MAP Kinase (Th180/Tyr182) (#9211), antibodies to p38—MAPK (#9212), and antibodies to phospho AKT (Ser473) were from Cell signaling technology, Danvers, Massachusetts, USA. FITC-dextran (Fluorescein isothiocyanate–dextran, 2x10^6^ kDa) and Anti-Protein Kinase-Cα (PKCα) were from Sigma-Aldrich, St. Louis, USA. Isolectin IB4-594 was from Alexa Fluor, Molecular probes, Eugene, USA. The antibody to Glial Fibrillary Acidic Protein (GFAP) was from Millipore, Billerica, Massachusetts, USA. Alexa Fluor^®^ 594 Donkey Anti-Mouse IgG (H+L) was from Life Technologies-Molecular Probes, Carlsbad, California, USA. Cy3-Affinipure Goat Anti-Rabbit IgG (H+L) was from Jackson ImmunoResearch Labratories, INC, West Grove, Pennsylvania, USA. The in situ cell death detection kit, TUNEL, was from Roche Diagnostics, Mannheim, Germany.

### Phosphorylation assays

Human umbilical vein derived endothelial cells (HUVEC) were isolated, and cultured as previously described [36] and grown to 90% confluence. The cells were incubated overnight in growth medium containing 2% FCS. FR-Sema3C/Fc (2 μg/ml) or vehicle was then added and after 10 min. at room temperature VEGF (30 ng/ml) or bFGF (5 ng/ml) or PDGF-BB (50 ng/ml) or HGF (50 ng/ml) or EGF (50 ng/ml) were added and after 10 more minutes the cells were lysed. ERK1/2, p38 MAPK, FAK^125^ and AKT phosphorylation levels were then determined essentially as previously described using western blot analysis followed by quantification of the intensity of stained bands using an Imagequant Las 4000 machine [37].

### Animals

C57 black mice were obtained from the Jackson Laboratory, USA.

### Induction and quantification of CNV and administration of therapeutics

C57 black mice at ages between 6–10 weeks were anesthetized by intramuscular ketamine hydrochloride injection at 80 mg/kg and xylazine at 16 mg/kg. For all procedures, 1% tropicamide and 2.5% phenylephrine hydrochloride were administered. After receiving anaesthesia and undergoing pupillary dilation, the animals were positioned before a slit lamp. The fundus was visualized using a microscope slide coverslip coated with 2.5% hypromellose ophthalmic demulcent solution which was held on the mouse cornea and served to subtract the optics of the cornea and lens for optimal view of the retina and spots of the laser lesions. Four laser burns were performed at equal distances around the optic discs of both eyes of C57 black mice using a Novus Spectra ophthalmic laser (Lumenis, Inc., Santa Clara, California, USA) to induce CNV. Burns were performed for 0.05 seconds at 250 mW. The burns were observed to produce an acute vapour bubble, indicating rupture of Bruch’s membrane.

FR-Sema3C/Fc (50 ng or 100 ng), aflibercept (5 μg) or vehicle alone were administered in a volume of 2 μl by intravitreal injection immediately after laser photocoagulation. The eyes of the mice were decompressed by inserting a 30G needle through the conjunctiva and sclera 1 mm behind the limbus. A UMP3 Micro injector equipped with a Nanofil syringe of 10 μL and 33G blunt needle (Word precision instruments, Sarasota, Florida, USA) was used for injection. The posterior segment was evaluated immediately after injection to confirm placement of the drug into the vitreous cavity and for ocular perfusion. After a week the animals were anesthetised. The mouse right atrium was cut and the vascular system perfused for 3 min with 0.5 ml/min of PBS through the left ventricle with assistance of a perfusion pump, followed by injection of 1.5 mL PBS containing 50 mg/mL fluorescein-labeled dextran. The eyes were removed, eye balls were enucleated carefully and the enucleated eyes fixed overnight in 4% para-formaldehyde (PFA) at 4°C. After rinsing in PBS, the cornea, iris and lens were excised, the eye cup was unfolded, and the neural retina peeled away from the underlying retinal pigment epithelium (RPE). The RPE/choroid/sclera complex was flat-mounted onto a slide with the RPE side facing up. A laser spot with green vessels was scored as CNV-positive, and a laser spot lacking green vessels was scored CNV-negative.Stained vessels were then visualized using a Zeiss Axio Imager M2 system equipped with Apotome, and attached to AxioCam and AxioVision cameras. Each laser lesion site was individually evaluated and photographed at 10 X magnification. The area of stained blood vessels that invaded laser burns was then measured using the free hand selection tool of the Axiovision 4.8 microscopy software and expressed in square micrometers (μm^2^). The mean area of invading blood vessels per laser burn was determined from 8 separate laser burns that were performed in both eyes. The means obtained from individual mice within an experimental group were then compared.

For Isolectin-IB_4_ staining animals were euthanized, eyes balls were enucleated and fixed overnight in 4% paraformaldehyde. Retinas were collected and the underlying retinal pigment epithelium (RPE) was exposed as described above. The retinas and the eye cups were incubated in PBS containing 0.5% triton for 5 h at room temperature. Eyecups and retinas were then incubated with alexa fluor 594 conjugated Isolectin–IB4 (10 μg/ml) essentially as described [9]. The eyecups and retinas were flat-mounted onto a slide and stained vessels visualized using a Nikon Eclipse E-600 fluorescent microscope and photographed. The area of stained blood vessels that invaded laser burns was then determined as described above.

### Optokinetic reflex (OKR) measurements

Visual function was assessed using a non-invasive OptoMotry optokinetic testing system (Cerebral Mechanics, Lethbridge, AB, Canada, USA), following the procedures described and validated previously [38]. Briefly, mice standing unrestrained on a central platform tracked a rotating grating with reflexive head movement behavior. Testing was initiated by projecting a grating of low spatial frequency (0.042 cycles/degree (c/d)), rotating clockwise for left eye measurement or counter clockwise for right eye measurement at 12 degrees/second at maximum 100% contrast. The threshold of maximum spatial frequency that the mouse could track was then determined for each eye.

### Electroretinogram (ERG) measurements

ERG measurements were conducted essentially as previously described [39]. After overnight dark adaptation, mice were anesthetized using ketamine (80 mg/kg) and xylazine (16 mg/kg). Eye drops (1% tropicamide and 2.5% phenylephrine HCl, and 1% proparacaine HCl) were used to dilate the pupils and to anesthetize the corneal surface. A pair of gold-wire contact lens electrodes was used to record ERGs from each corneal surface. These active leads were referenced to a needle electrode placed subcutaneously between the eyes. A second needle electrode placed in the tail served as the ground lead. ERGs were recorded using an LKC (Gaithersburg, MD, USA) UTAS E-3000 signal averaging system. Responses evoked by ganzfeld strobe flash stimuli were band-pass filtered (0.03–1000 Hz), amplified, averaged and stored. In each recording session, we began with scotopic ERG responses to low-luminance stimuli and increased flash luminance as the session proceeded. Stimuli were presented in order of increasing luminance and ranged in flash luminance from −3.3 to 1.1 log cd s/m^2^. After the recordings of scotopic ERG responses were complete, cone ERGs were isolated for measuring photopic ERG responses by superimposing stimuli upon a steady adapting field (20 cd/m^2^). Flash luminance ranged from −0.8 to 1.2 log cd s/m^2^. All procedures were performed under dim red light, and the mice were kept on a heating pad to maintain a constant body temperature during the ERG recording. The a-wave amplitude was measured from baseline to the trough of the a-wave, whereas the b-wave was measured from the trough of the a-wave to the peak of the b-wave.

### TUNEL

TUNEL was performed essentially as previously described [40]. Retinas treated with DNase were used as a positive control.

### Histology and immunocytochemistry

Eyes were dissected from euthanized mice, fixed in 4% paraformaldehyde (PFA) overnight at 4°C, embedded in paraffin and sectioned into 7 μm sections. haematoxylin and eosin (H&E) staining was performed as previously described [41]. For immunohistochemistry eye cups were fixed in 4% PFA at 4°C for 1 hour followed by consecutive 1 hour incubations in 5%, 10% and 15% sucrose followed by overnight incubation in 20% sucrose at 4°C. The eyecup were then embedded in OCT and sectioned into 15-μm thick sections along the vertical meridian using a cryostat. Sections were washed 3 times for 5 min in PBS pH 7.4, and incubated in non-immune serum (4% Goat serum, 0.01% Tween 20, PBS 0.1 M). Sections were then incubated overnight at 4°C with anti- Glial Fibrillary Acidic Protein (GFAP) antibodies at a 1:100 dilution, or with anti-PKCα at a 1:100, rinsed three times in PBS and incubated respectively for 2h with a donkey anti mouse Alexafluor 594 labeled antibody or with Cy3 conjugated goat anti-rabbit antibody followed by two washes with PBS. Slides were counterstained with DAPI, mounted, and photographed using a Nikon Eclipse E-600 fluorescent microscope.

### Statistical analysis

The one tailed Mann-Whitney test was used to assess the effects of FR-sema3C on growth factor induced ERK1/2 phosphorylation. To assess the effects of FR-sema3C on CNV induced by laser photocoagulation, the mean area of invading blood vessels per laser burn was determined from 8 separate laser burns that were performed in both eyes. The means obtained from individual mice within an experimental group were then compared and evaluated for statistical significance using one way ANOVA followed by Bonferroni’s multiple comparison post test. The statistical significance of the OKR and ERG test results was assessed using student’s t-test. The following designations were used in the figures: *: p<0.05, **: p<0.01 and non-specific: ns.

### Adherence to ARVO regulations

This study adhered to ARVO Statement for the use of Animals in ophthalmic and vision research and was approved by the committee for animal experiments at the Faculty of Medicine of the Technion and by the institutional animal care and use committee, the animal care committee at the University of Louisville.

## Results

### FR-sema3C inhibits VEGF and PDGF-BB signal transduction in endothelial cells

**Fig 1:**
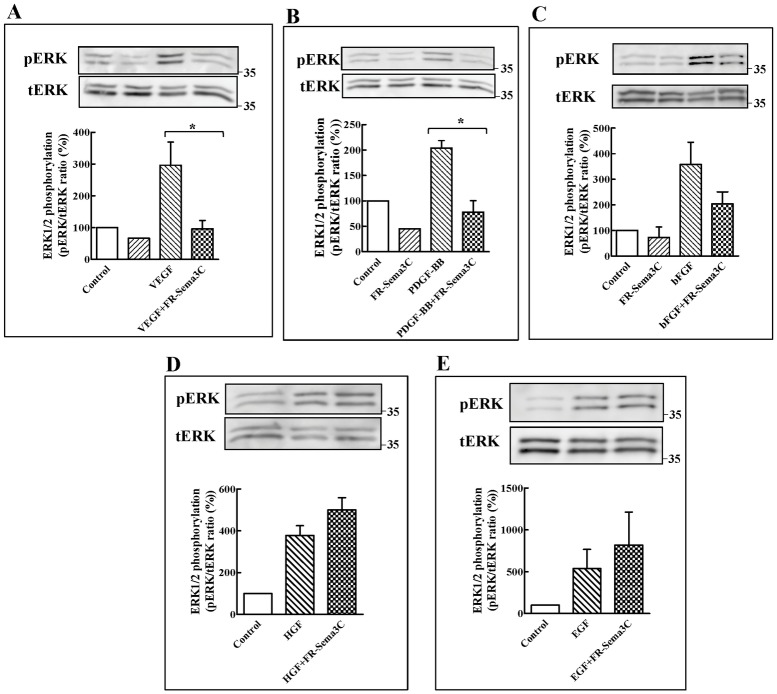
FR-sema3C inhibits VEGF and PDGF-BB induced phosphorylation of ERK1/2 in endothelial cells. (A) HUVEC were stimulated or not with VEGF (30 ng/ml) in the presence of elution buffer (100 mM glycine, 24 mM Tris, pH-7.2) or FR-sema3C/Fc (2 μg/ml). After 10 min. at room temperature the cells were lysed and ERK1/2 phosphorylation was determined by western blot as described. Shown is a representative experiment out of three that gave similar results. Below is shown a histogram depicting the ratio between the intensity of the respective phospho-ERK1/2 bands and the total ERK bands. (B) HUVEC were stimulated with PDGF-BB (50 ng/ml) in the presence or absence of FR-sema3C/Fc (2 μg/ml). ERK1/2 phosphorylation was determined and quantified as described under A. Shown is a representative experiment out of three that gave similar results. (C) HUVEC were stimulated with bFGF (5 ng/ml) in the presence or absence of FR-sema3C/Fc (2 μg/ml). ERK1/2 phosphorylation was determined and quantified as described under A. Shown is a representative experiment out of three that gave similar results. (D) HUVEC were stimulated with HGF (50 ng/ml) in the presence or absence of FR-sema3C/Fc (2 μg/ml). ERK1/2 phosphorylation was determined and quantified as described under A. Shown is a representative experiment out of three that gave similar results. (E) HUVEC were stimulated with EGF (50 ng/ml) in the presence or absence of FR-sema3C/Fc (2 μg/ml). ERK1/2 phosphorylation was determined and quantified as described under A. Shown is a representative experiment out of three that gave similar results. Statistical significance was evaluated using the one tailed Mann-Whitney test. Error bars represent the standard error of the mean. *: p<0.05.

We have previously determined that FR-sema3C inhibits signal transduction by the angiogenic and lymphangiogenic factor VEGF-C and that it inhibits tumor angiogenesis [30,42]. Sema3C binds to the neuropilin-1 and neuropilin-2 receptors which also function as essential co-receptors for several VEGF family members and is thus expected to inhibit VEGF signal transduction [43,44]. However, several additional angiogenic factors such as platelet derived growth factor (PDGF), hepatocyte growth factor (HGF), epidermal growth factor (EGF) and basic fibroblast growth factor (bFGF) have also been reported to play possible roles in AMD [5,6,45–47]. Interestingly, signal transduction by these factors was also found to be affected by neuropilins, either because neuropilins serve as binding receptors to these factors or because their receptors form complexes with neuropilins [48–51]. Activation of plexins by class-3 semaphorins also activates inhibitory signalling cascades that can inhibit signal transduction induced by angiogenic factors even when the semaphorins do not compete with these factors for binding to neuropilins [37]. Since these angiogenic factors all activate the *ras* signalling cascade we determined if FR-sema3C can inhibit in cultured human umbilical vein derived endothelial cells (HUVEC) ERK1/2 phosphorylation induced by these factors as a proof of concept in order to determine if FR-sema3C has the potential to inhibit their activity in AMD. To this end we used purified FR-sema3C that was epitope tagged at the C-terminal with an Fc tag (FR-sema3C/Fc) as previously described [30]. FR-sema3C/Fc inhibited VEGF induced signal transduction as manifested by its ability to inhibit VEGF induced phosphorylation of ERK1/2 (Fig 1A), P38 Map Kinase, AKT and FAK^125^ (S1A–S1C Fig). FR-sema3C also inhibited PDGF-BB (Fig 1B) induced activation of ERK1/2 in endothelial cells and also partially inhibited bFGF induced activation of ERK1/2 in three independent experiments although the inhibition did not reach statistical significance (Fig 1C). However, FR-sema3C failed to inhibit ERK1/2 phosphorylation induced by hepatocyte growth factor (HGF) and epidermal growth factor (EGF) although HGF is known to transduce signals using the sema3C receptor neuropilin-1 (Fig 1D and 1E) [49]. These observations suggest that FR-Sema3C may have a wider range of anti-angiogenic activity as compared with currently used drugs such as bevacizumab or ranibizumab which selectively target VEGF. However, aflibercept was also able to inhibit PDGF-BB induced phosphorylation of ERK1/2 but not bFGF induced phosphorylation of ERK1/2 (S1D and S1E Fig).

### FR-sema3C inhibits laser induced CNV as efficiently as aflibercept

**Fig 2:**
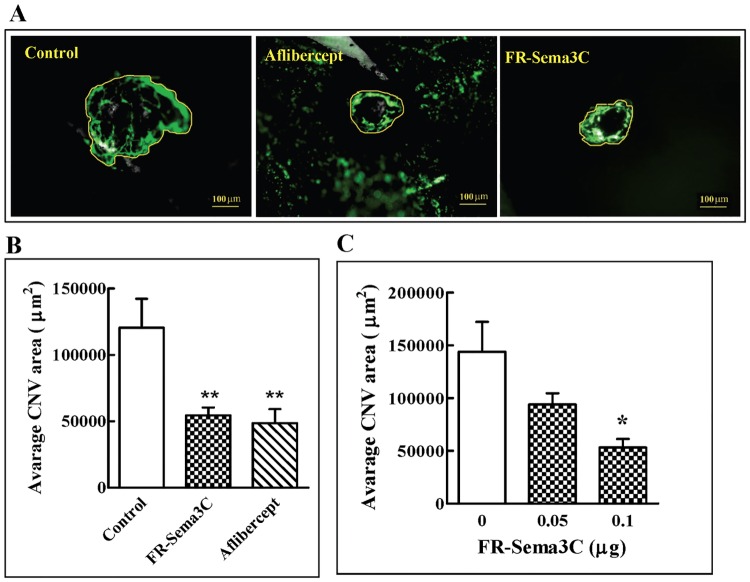
FR-Sema3C inhibits CNV induced by laser photocoagulation. (A) Four laser burns were made around the optic nerve in each eye of C57 black mice (6 mice per group). Eyes were injected immediately after with FR-sema3C/Fc (0.1 μg) or with aflibercept (5 μg) in a 2 μl volume, or with an equal volume of vehicle (100 mM glycine, 24 mM Tris, pH-7.2) (Control) as described. The RPE/choroid/sclera complex was excised after a week following injection of FITC-dextran into the circulation. Shown are representative fluorescent photographs of blood vessels invading laser burns from control, aflibercept and FR-Sema3C/Fc treated eyes. The area representing the CNV area measured in B is encircled by a yellow line. (B) Quantification of the area of stained blood vessels that invaded laser burns in the experiment described under A was performed as described in materials and methods. The means obtained from individual mice within an experimental group were then compared and evaluated for statistical significance using one way ANOVA followed by Bonferroni’s multiple comparison post test. (C) The effect of increasing FR-Sema3C/Fc concentrations on CNV was evaluated as described. To assess the effects of FR-sema3C on CNV induced by laser photocoagulation, the mean area of invading blood vessels per laser burn was determined from 8 separate laser burns that were performed in both eyes. The means obtained from individual mice within an experimental group (4 mice) were then compared and evaluated for statistical significance using one way ANOVA followed by Bonferroni’s multiple comparison post test. Error bars represent the standard error of the mean. *: p<0.05, **: p<0.01.

In order to find out if FR-Sema3C does indeed possess potential as a possible candidate drug for the treatment of exudative AMD, we determined if injection of FR-Sema3C/Fc into the vitreal chamber of C57 black mouse eyes can inhibit CNV induced by laser photocoagulation [52]. A single bolus intravitreal injection of 100 ng FR-Sema3C/Fc [30] was performed immediately after laser photocoagulation. FR-sema3C inhibited CNV significantly as determined by the comparison of the mean areas of invading blood vessels per laser burn in individual vehicle treated mice (120604 +/- 21727 μm^2^), with the mean areas of invading blood vessels per laser burn in individual FR-Sema3C/Fc treated mice (54518 +/- 5858 μm^2^) (p<0.01) a week after induction of CNV (Fig 2A and 2B). The inhibition produced by FR-sema3C/Fc was comparable to the inhibition produced by a single bolus injection of 5 μg aflibercept [53] which is probably the most effective drug currently used to treat exudative AMD (Fig 2A and 2B). We also treated mice with lower concentrations of FR-sema3C but although 50 ng of FR-sema3C consistently inhibited CNV, the inhibition failed to reach statistical significance (Fig 2C). We have also compared the effects of FR-sema3C/Fc on laser photocoagulation induced CNV in the mouse model with those of sema3C/Fc. Both FR-sema3C/Fc and the commercial sema3C/Fc had a similar molecular mass of about 130 kDa and there were no visible proteolytic fragments (S2A Fig). Both FR-sema3C and sema3C inhibited CNV similarly and although sema3C seemed a bit less active the difference was not statistically significant (S2B Fig). We also determined if purified p65-Sema3C/myc (S2A Fig) [30] affects laser photocoagulation induced CNV. However, as expected, p65-Sema3C did not inhibit laser photocoagulation induced CNV (S2C Fig).

### FR-sema3C does not impair the function of the retina

**Fig 3:**
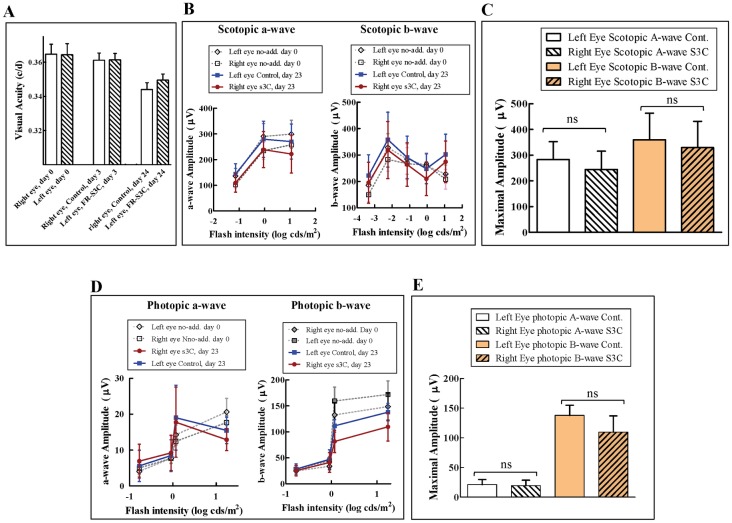
The function of the retina is not compromised after a single bolus intravitreal injection of FR-sema3C. (A) Six mice were injected with 2 μL of vehicle (100 mM glycine, 24 mM Tris, pH-7.2) in their right eyes and with 2 μl of FR-sema3C/Fc (0.1 μg) in their left eyes. Visual function was measured by OKR tests performed on day-3 and 24 after intravitreal injection as described in materials and methods, and compared to the Visual function of both eyes prior to injection (day 0). (B and D) Vehicle alone (control) was injected into the vitreal cavities of the left eyes of six mice and FR-sema3C/Fc (s3c) (0.1 μg) was injected into the vitreal cavities of their right eyes as described under A. Scotopic (B) and photopic (D) ERG responses of a- and b-wave amplitudes were measured 23 days after injection as described in materials and methods and compared to the scotopic and photopic ERG responses of both eyes prior to injection (day 0). (C and E). The average maximal ERG response amplitude of scotopic (C) and photopic (E) a-waves and b-waves of eyes treated with vehicle alone (Cont.) or with FR-sema3C/Fc (S3C) 23 days after FR-sema3C/Fc injection. Error bars represent the standard error of the mean. Statistical significance was evaluated using student’s t-test. ns: non-specific.

In order to determine if FR-sema3C/Fc has any detrimental effects on vision by itself, we injected a single bolus of 100 ng FR-sema3C/Fc into the vitreous of left eyes of mice and a similar volume of vehicle into their right eyes. The effects of the injections on visual acuity were determined prior to the injections as well as three days and twenty four days after injection using the OKR test [38]. After 3 days there was no difference between control eyes and eyes injected with FR-sema3C/Fc nor was there loss of visual acuity as compared with the measurements done in both groups of mice prior to injection (Fig 3A). After 24 days there was still no difference in visual acuity between control eyes and eyes injected with FR-sema3C/Fc although there was some minor deterioration of visual acuity in both control and FR-sema3C/Fc treated eyes which was probably due to non-specific effects of the injection procedure (Fig 3A). These results suggest that FR-sema3C/Fc does not hamper visual acuity by itself (Fig 3A).

In order to determine if FR-sema3C/Fc exerts detrimental effects on the response of the retina to light stimuli, we also performed ERG tests before injection and 23 days after injection of a single 100 ng bolus of FR-sema3C/Fc into the vitreous of the right eyes of healthy mice and compared the responses with the responses of the left eyes which were injected with an equal volume of vehicle (Fig 3B and 3D). In this test too we did not observe a significant difference between scotopic and photopic ERG responses of eyes that received vehicle alone or FR-sema3C/Fc after 23 days (Fig 3B and 3D). There was also no statistically significant difference between the average maximal EGR response amplitudes of scotopic and photopic a and b-waves of eyes treated with FR-sema3C/Fc and the average maximal EGR response amplitudes of scotopic and photopic a and b-waves of eyes treated with vehicle alone after 23 days (Fig 3C and 3E).

**Fig 4:**
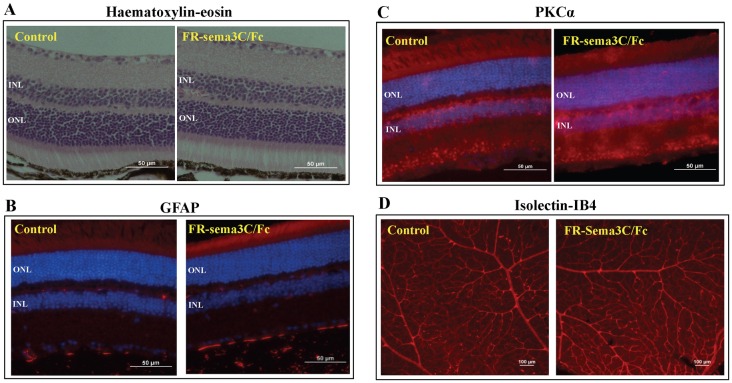
Injection of FR-sema3C does not affect retinal morphology. (A) Shown are representative photographs of haematoxylin-eosin stained transverse sections from a retina derived from the eye of a mouse injected with vehicle alone (Control) or from an eye injected with FR-sema3C/Fc after 23 days. (B) Shown are representative photographs of a GFAP stained retina derived from the eye of a mouse injected with vehicle (control) or from an eye injected with FR-sema3C/Fc after 23 days. Both retinas were devoid of GFAP labeling in Müller cells. (C) Shown are representative photographs of PKCα stained retinas derived from the eye of a mouse injected with vehicle alone (control) or from an eye injected with FR-sema3C/Fc 23 days previously. (D) Shown are representative photographs of isolectin-IB_4_ stained retinal blood vessels. The retinas were collected after a week following intravitreal injection with FR-Sema3C/Fc (0.1 μg) or Vehicle alone (control). O.N.L-Outer nuclear layer, I.N.L-Inner nuclear layer.

We also compared haematoxylin-eosin stained histological sections of retinas derived from six vehicle and six FR-sema3C/Fc treated eyes after 23 days. However, in none of the FR-sema3C/Fc treated retinas could we detect any significant differences when compared to retinas injected with vehicle alone (Fig 4A). We have also compared the expression pattern of GFAP in vehicle and FR-sema3C/Fc treated retinas after 23 days. Following retinal insults causing functional and structural damage, GFAP is frequently expressed in retinal Müller cells [54,55]. However, we could not detect any GFAP expression in retinal Müller cells in any of the six FR-sema3C/Fc treated retinas or in any of the vehicle injected eyes, indicating that FR-sema3C/Fc injection is not harmful to the retina (Fig 4B).

PKC immunoreactivity is used to identify rod bipolar cells in the vertebrate retina [56]. Retinal staining for PKC immunoreactivity of retinas from eyes treated with FR-sema3C/Fc 23 days post injection did not reveal any differences between FR-sema3C treated and control eyes (Fig 4C). Furthermore, there was no discernible difference between the morphology of retinal blood vessels of eyes injected with FR-sema3C and eyes that were treated with vehicle only (Fig 4D). Likewise, injection of wild type sema3C also had not effect of the morphology of mature retinal blood vessels (S3B Fig) as previously reported [23]. Injection of FR-sema3C also failed to induce apoptosis in the retina as determined using the TUNEL assay (S3A Fig) nor did the treatment affect the integrity of the retinal pigment epithelial cells layer (S3C Fig).

## Discussion

We show here that FR-Sema3C, a full length point mutated sema3C resistant to cleavage by FPPC inhibits VEGF as well as PDGF-BB and to some extent also bFGF induced signal transduction in cultured endothelial cells as well as laser photocoagulation induced CNV in a mouse AMD model. We have used FR-sema3C rather than wild type sema3C even though both function as effective inhibitors of angiogenesis [23,30], because previous observations we have made suggest that p65-Sema3C, the main FPPC cleavage product derived from sema3C, functions, unlike full length sema3C, as a survival/mitogenic factor for some tumor cell types [30]. Because FPPC expression is widespread, we reasoned that use of FR-sema3C may involve a lesser danger of untoward side effects even though FR-sema3C and wild type sema3C inhibited similarly laser photocoagulation induced CNV in the mouse model. VEGF, PDGF-BB and bFGF have been reported to be involved in AMD [5,6,57]. Signaling by both VEGF and PDGF-BB is strongly dependent on the presence of neuropilins [15,16,48,58] and it is possible that FR-sema3C, which binds to both neuropilins, inhibits signal transduction by VEGF and PDGF-BB because it competes with these factors for binding to neuropilins as we have previously demonstrated in the case of VEGF-C [30]. However, this may not represent the only mechanism by which FR-sema3C inhibits CNV. We have shown previously that some class-3 semaphorins such as sema3A and sema3F can inhibit signal transduction by VEGF and bFGF even though these semaphorins do not inhibit bFGF and VEGF induced auto-phosphorylation of VEGF and bFGF receptors. These observations suggest that these semaphorins activate an inhibitory signaling cascade mediated by their neuropilin/plexin receptors which counteracts the activity of these growth factors without affecting their binding to their cell surface receptors [37]. It is thus also possible that part of the inhibitory effect of FR-sema3C is not due to competition with angiogenic factors for binding to neuropilins but rather due to the activation of inhibitory intracellular signaling pathways. Notably, HGF and EGF induced activation of ERK1/2 was not inhibited by FR-sema3C even though it is well established that HGF binds to neuropilins and its activity is enhanced by neuropilins [49]. Thus, FR-sema3C seems to function by a mechanism that may not involve competition with VEGF for binding to shared receptors and may possess a wider range of anti-angiogenic activity as compared with that of VEGF inhibitors such as bevacizumab or ranibizumab.

Currently 23–30% of exudative AMD patients either do not benefit from treatment with VEGF inhibitors or further loose vision as assessed in the ANCHOR and CATT clinical trials [7]. Furthermore, More than 50% of patients treated with anti-VEGF medications demonstrate persistent sub-macular fluid on OCT, an indicator of active disease. Inefficacy of anti-VEGF therapy may result from the treatment's inability to target established vascular lesions or because these treatments cannot prevent the emergence of new lesions driven by VEGF-independent angiogenesis pathways such as ECM-induced, neuropilin-1 dependent angiogenesis [59], or angiogenesis driven by angiogenic factors different from VEGF. Our observations suggest that because FR-Sema3C inhibits the activities of VEGF-C [30], PDGF and bFGF in addition to VEGF, it may possibly benefit this large group of AMD patients who do not respond well to the currently used VEGF binding drugs such as bevacizumab (Avastin^™^), aflibercept (Eylea^™^) and ranibizumab (Lucentis^™^) which sequestrate and trap VEGF. In AMD the permeabilization of blood vessels by VEGF is a major factor that contributes to macular damage along with the de-regulated growth of blood vessels from the choroid. We have not yet determined if FR-sema3C can inhibit the extravasation of fluids from blood vessels that accompanies CNV and this will need to be examined in the future.

In the laser induced CNV mouse model FR-Sema3C inhibited CNV as effectively as aflibercept at a concentration that was 50 fold lower than the effective aflibercept concentration [60]. Importantly, FR-sema3C did not compromise retinal structure as assessed by histological and immunochemical examination nor did it affect retinal function as assessed by functional OKR and ERG tests. However, it is possible that the histological and functional assays we performed may not be able to detect subtle defects resulting from treatment with FR-sema3C, and in addition it is likely that the human responses to FR-sema3C may differ from the responses of mice.

The mechanism of action of FR-sema3C differs from that of the current mainstay, VEGF-A targeting drugs, as it seem to function by activation of signal transduction involving neuropilins and plexin-D1 [23,30]. Sema3C receptors are expressed in AMD [22,57,61] and there is evidence suggesting that plexin-D1 and neuropilin1 are up-regulated during CNV [22,62]. Thus, FR-sema3C may perhaps be of benefit for those patients that do not respond well to current treatments with VEGF inhibitors. In mice FR-sema3C seems safe as it did not compromise retinal function. Use of AMD models in larger animals will be required in order to obtain more information on the advantages and disadvantages of FR-Sema3C as a candidate drug for the treatment of AMD. However, the lack of an optimal animal model that recapitulates the slow events that eventually lead to the development of the exudative form of AMD in humans seems to suggest that such questions may not be resolved without clinical trials.

## Supporting Information

S1 Fig FR-sema3C inhibits in endothelial cells VEGF induced phosphorylation of P38 MAP-Kinase, AKT and FAK^125^. (A) HUVEC were stimulated or not with VEGF (30 ng/ml) in the presence of elution buffer (100 mM glycine, 24 mM Tris, pH-7.2) or FR-sema3C/Fc (2 μg/ml). After 10 min. at room temperature the cells were lysed and p38 MAPK phosphorylation on T180 and Y182 was determined using western blot analysis as described in materials and methods. Shown is a representative experiment out of three that produced similar results. Below is shown a histogram depicting the ratio between the intensity of the respective pT180/Y182-p38 MAPK bands and the total p38 MAPK bands. (B) HUVEC were stimulated as described under A. FAK^125^ phosphorylation was determined and quantified as described in materials and methods. Below is shown a histogram depicting the ratio between the intensity of the phosphorylated FAK^125^ pY397 bands and total FAK. Shown is a representative experiment out of two that produced similar results. (C) HUVEC were stimulated as described under A. AKT phosphorylation was determined and quantified as described in materials and methods. Shown is a representative experiment out of two that gave similar results. A histogram depicting the ratio between the intensity of the respective pS473-AKT bands and the total AKT bands is shown below. (D) HUVEC were stimulated or not with PDGF-BB (50 ng/ml) in the presence or absence of Aflibercept (2 μg/ml). Shown is a representative experiment out of three that gave similar results. ERK1/2 phosphorylation was determined and quantified as described in materials and methods. (E) HUVEC were stimulated or not with bFGF (5 ng/ml) in the presence or absence of Aflibercept (2 μg/ml). ERK1/2 phosphorylation was determined and quantified as described in materials and methods. Shown is a representative experiment out of two that gave similar results. Below is shown a histogram depicting the ratio between the intensity of the respective phospho-ERK1/2 bands and the total ERK1/2 bands.
(TIFF)

S2 Fig Comparison of the effects of sema3C, FR-sema3C and p65-Sema3C on laser photocoagulation induced CNV. (A) FR-Sema3C, Sema3C and p65-Sema3C were resolved on an 8% SDS/PAGE gel and subjected to western blot analysis using antibodies directed against the N-terminal of Sema3C. (B) Four laser burns were made around the optic nerve in each eye of C57 black mice (3 mice per group). Eyes were injected immediately after with FR-sema3C/Fc (0.1 μg) or with Sema3C (0.1 μg) in a 2 μl volume, or with an equal volume of vehicle (100 mM glycine, 24 mM Tris, pH-7.2) (Control) as described. The RPE/choroid/sclera complex was excised after a week following injection of FITC-dextran into the circulation. Quantification of the area of stained blood vessels that invaded laser burns was performed as described. Statistical analysis was performed as described in Fig 2. Error bars represent the standard error of the mean. *: p<0.05. (C) Four laser burns were made around the optic nerve in each eye of the C57 black mice (3 mice per group). Eyes were injected immediately after with p65-Sema3C (0.1 μg) in a 2 μl volume, or with an equal volume of vehicle (Control) as described above. The RPE/choroid/sclera complex was excised after a week following injection of FITC-dextran into the circulation. Quantification of the area of stained blood vessels that invaded laser burns and statistical analysis were performed as described above.
(TIFF)

S3 Fig Images of retinas and flat mounted choroid before and after treatment. (A) Mice were intravitreally injected with FR-Sema3C (0.1 μg) or with Sema3C (0.1 μg) or with Vehicle alone (control). Retinas were collected after a week and apoptotic cells were visualized by TUNEL staining. The bright dots represent the TUNEL-stained pyknotic bodies. For positive control, retinas were treated with 2,500 units/ml of DNAse I for 10 min. Shown are representative images out of four retinas that were assayed in each group. (B) Mice were intravitreally injected with Sema3C (0.1 μg) or Vehicle alone (control), and retinas were collected after a week as described. Retinal vessels were visualized following staining with Isolectin-IB4. (C) Representative images of flat-mounted choroid after isolectin-IB_4_ staining a week after laser photocoagulation in comparison with FR-Sema3C/Fc treated eyes that were not subjected to laser photocoagulation. O.N, optic nerve.
(TIFF)
