# Supplementary figures and images for: A Sema3C Mutant Resistant to Cleavage by Furin (FR-Sema3C) Inhibits Choroidal Neovascularization
Source: PLoS One. 2016 Dec 30;11(12):e0168122. doi: 10.1371/journal.pone.0168122 (PMC5201251; doi:10.1371/journal.pone.0168122)

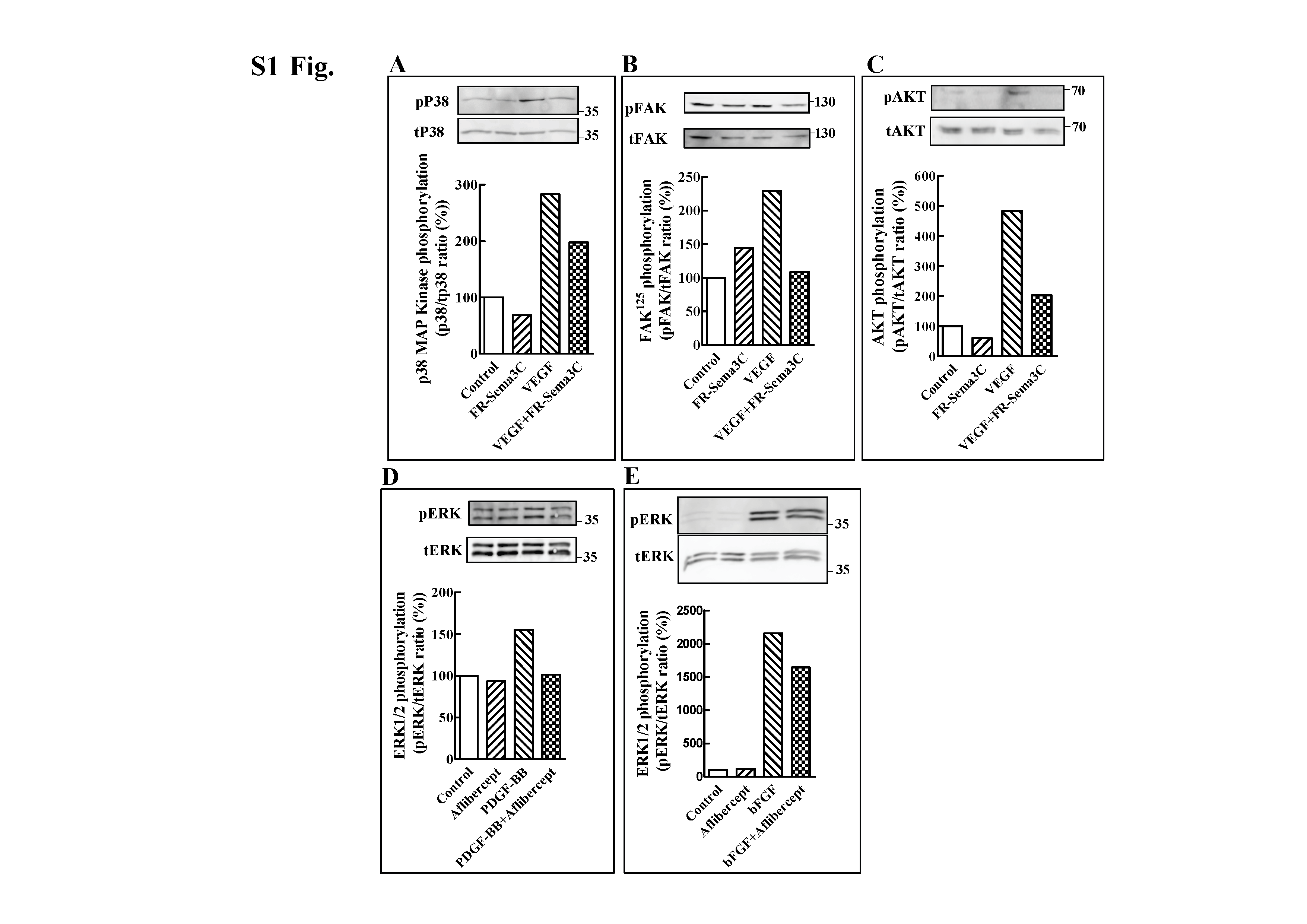

Supplement: S1 Fig — (A) HUVEC were stimulated or not with VEGF (30 ng/ml) in the presence of elution buffer (100 mM glycine, 24 mM Tris, pH-7.2) or FR-sema3C/Fc (2 μg/ml). After 10 min. at room temperature the cells were lysed and p38 MAPK phosphorylation on T180 and Y182 was determined using western blot analysis as described in materials and methods. Shown is a representative experiment out of three that produced similar results. Below is shown a histogram depicting the ratio between the intensity of the respective pT180/Y182-p38 MAPK bands and the total p38 MAPK bands. (B) HUVEC were stimulated as described under A. FAK125 phosphorylation was determined and quantified as described in materials and methods. Below is shown a histogram depicting the ratio between the intensity of the phosphorylated FAK125 pY397 bands and total FAK. Shown is a representative experiment out of two that produced similar results. (C) HUVEC were stimulated as described under A. AKT phosphorylation was determined and quantified as described in materials and methods. Shown is a representative experiment out of two that gave similar results. A histogram depicting the ratio between the intensity of the respective pS473-AKT bands and the total AKT bands is shown below. (D) HUVEC were stimulated or not with PDGF-BB (50 ng/ml) in the presence or absence of Aflibercept (2 μg/ml). Shown is a representative experiment out of three that gave similar results. ERK1/2 phosphorylation was determined and quantified as described in materials and methods. (E) HUVEC were stimulated or not with bFGF (5 ng/ml) in the presence or absence of Aflibercept (2 μg/ml). ERK1/2 phosphorylation was determined and quantified as described in materials and methods. Shown is a representative experiment out of two that gave similar results. Below is shown a histogram depicting the ratio between the intensity of the respective phospho-ERK1/2 bands and the total ERK1/2 bands. (TIFF) [file pone.0168122.s001.tiff]

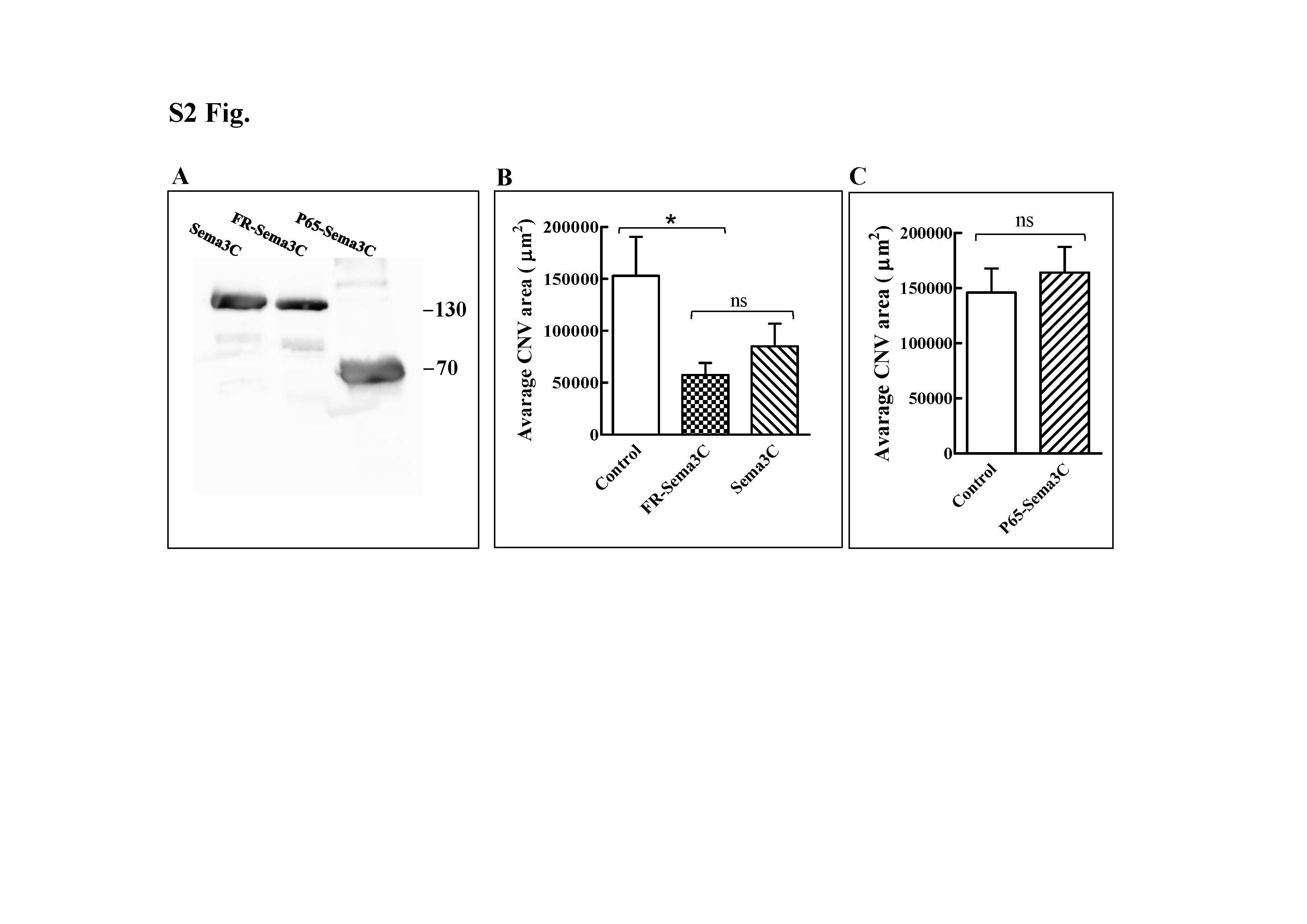

Supplement: S2 Fig — (A) FR-Sema3C, Sema3C and p65-Sema3C were resolved on an 8% SDS/PAGE gel and subjected to western blot analysis using antibodies directed against the N-terminal of Sema3C. (B) Four laser burns were made around the optic nerve in each eye of C57 black mice (3 mice per group). Eyes were injected immediately after with FR-sema3C/Fc (0.1 μg) or with Sema3C (0.1 μg) in a 2 μl volume, or with an equal volume of vehicle (100 mM glycine, 24 mM Tris, pH-7.2) (Control) as described. The RPE/choroid/sclera complex was excised after a week following injection of FITC-dextran into the circulation. Quantification of the area of stained blood vessels that invaded laser burns was performed as described. Statistical analysis was performed as described in Fig 2. Error bars represent the standard error of the mean. *: p<0.05. (C) Four laser burns were made around the optic nerve in each eye of the C57 black mice (3 mice per group). Eyes were injected immediately after with p65-Sema3C (0.1 μg) in a 2 μl volume, or with an equal volume of vehicle (Control) as described above. The RPE/choroid/sclera complex was excised after a week following injection of FITC-dextran into the circulation. Quantification of the area of stained blood vessels that invaded laser burns and statistical analysis were performed as described above. (TIFF) [file pone.0168122.s002.tiff]

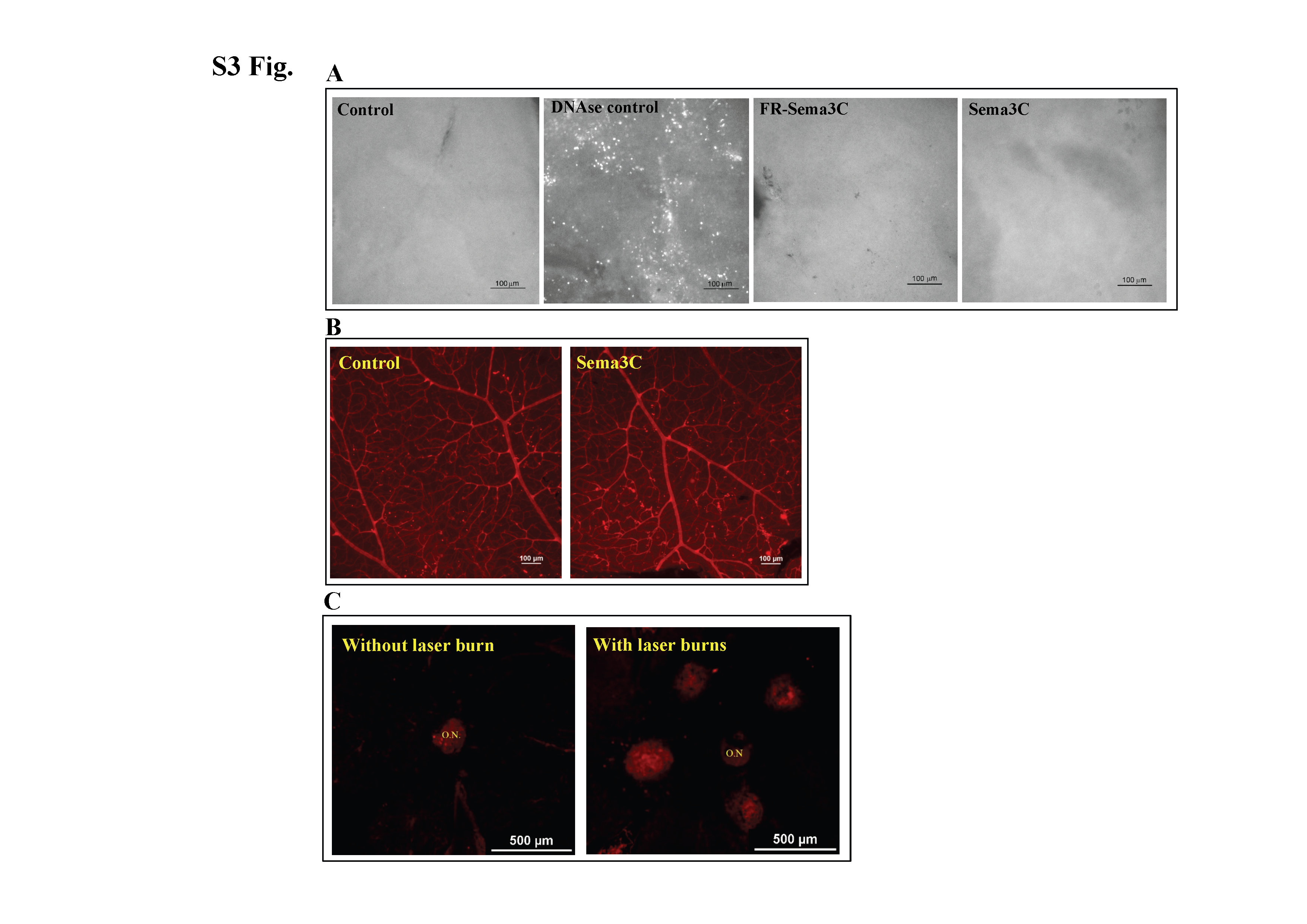

Supplement: S3 Fig — (A) Mice were intravitreally injected with FR-Sema3C (0.1 μg) or with Sema3C (0.1 μg) or with Vehicle alone (control). Retinas were collected after a week and apoptotic cells were visualized by TUNEL staining. The bright dots represent the TUNEL-stained pyknotic bodies. For positive control, retinas were treated with 2,500 units/ml of DNAse I for 10 min. Shown are representative images out of four retinas that were assayed in each group. (B) Mice were intravitreally injected with Sema3C (0.1 μg) or Vehicle alone (control), and retinas were collected after a week as described. Retinal vessels were visualized following staining with Isolectin-IB4. (C) Representative images of flat-mounted choroid after isolectin-IB4 staining a week after laser photocoagulation in comparison with FR-Sema3C/Fc treated eyes that were not subjected to laser photocoagulation. O.N, optic nerve. (TIFF) [file pone.0168122.s003.tiff]
